# Role of FUT8 expression in clinicopathology and patient survival for various malignant tumor types: a systematic review and meta-analysis

**DOI:** 10.18632/aging.202239

**Published:** 2020-12-11

**Authors:** Minxing Ma, Guoxiong Han, Yi Wang, Ziyan Zhao, Feng Guan, Xiang Li

**Affiliations:** 1 Key Laboratory of Resource Biology and Biotechnology in Western China, Ministry of Education, Institute of Hematology, School of Medicine, Northwest University, Xi’an, China; 2 Department of Oncology, The Fifth People’s Hospital of Qinghai Province, Xining, China; 3 Department of Hematology, Provincial People’s Hospital, Xi’an, Shaanxi, China; 4 Joint International Research Laboratory of Glycobiology and Medicinal Chemistry, College of Life Science, Northwest University, Xi’an, China

**Keywords:** fucosyltransferase 8, malignant tumor, prognosis, microarray, meta-analysis

## Abstract

Dysregulation of α(1,6)-fucosyltransferase (FUT8) plays significant roles in development of a variety of malignant tumor types. We collected as many relevant articles and microarray datasets as possible to assess the prognostic value of FUT8 expression in malignant tumors. For this purpose, we systematically searched PubMed, Embase, Web of Science, Springer, Chinese National Knowledge Infrastructure (CNKI), and Wan Fang, and eventually identified 7 articles and 35 microarray datasets (involving 6124 patients and 10 tumor types) for inclusion in meta-analysis. In each tumor type, FUT8 expression showed significant (p< 0.05) correlation with one or more clinicopathological parameters; these included patient gender, molecular subgroup, histological grade, TNM stage, estrogen receptor, progesterone receptor, and recurrence status. In regard to survival prognosis, FUT8 expression level was associated with overall survival in non-small cell lung cancer (NSCLC), breast cancer, diffuse large B cell lymphoma, gastric cancer, and glioma. FUT8 expression was also correlated with disease-free survival in NSCLC, breast cancer, and colorectal cancer, and with relapse-free survival in pancreatic ductal adenocarcinoma. For most tumor types, survival prognosis of patients with high FUT8 expression was related primarily to clinical features such as gender, tumor stage, age, and pathological category. Our systematic review and meta-analysis confirmed the association of FUT8 with clinicopathological features and patient survival rates for numerous malignant tumor types. Verification of prognostic value of FUT8 in these tumor types will require a large-scale study using standardized methods of detection and analysis.

## INTRODUCTION

Traditionally, in most common cancers, including breast cancer (BC), clinicopathological features (*e.g.*, tumor size, lymph node status, TNM stage, histological grade, hormone receptor status, human epidermal growth factor receptor 2 [HER-2] amplification) are used to predict patient outcome [1]. Biomarkers such as tumor-associated macrophages (TAMs), microRNAs, matrix metalloproteinases (MMPs), retinoic acid receptor a (RARA), and Ki-67 are also useful in predicting prognosis of certain cancers (*e.g.*, colon cancer, gastric cancer, acute promyelocytic leukemia) [2–6]. There has been increasing research focus on protein glycosylation and related glycosyltransferases as prognostic biomarkers in various human cancers [7–9].

Glycosylation (attachment of glycans to proteins or other organic molecules) is a common posttranslational modification in all organisms. Aberrant glycosylation is a characteristic phenomenon in carcinogenesis, plays essential roles in specific steps of tumor development [10], and directly promotes tumor progression and metastasis [11–17]. Glycosylation is mediated by enzymatic activities of glycosyltransferases and glycosidases in glycoproteins and/or lipids. Alterations of glycosyltransferase expression are associated with both pro-metastatic and metastasis-suppressing functions [12].

Fucosyltransferase 8 (FUT8) is an α(1,6)-fucosyltransferase responsible for addition of fucose to asparagine-linked N-acetylglucosamine (GlcNAc) moieties, a common feature of N-linked glycan core structures [18]. Aberrant fucosylated glycan structures and associated glycosyltransferases have been observed in development of various cancers [19]; *e.g.*, increased core fucosylation in BC [20], non-small cell lung cancer (NSCLC) [21], ovarian cancer [22], colon cancer [23], prostate cancer [24], and melanoma [25]. A positive feedback mechanism of FUT8-mediated receptor core fucosylation was recently shown to enhance TGF-β signaling and epithelial-mesenchymal transition (EMT), thus promoting BC cell invasion and metastasis; such fucosylation in BC patients is a potential diagnostic/ prognostic biomarker, or therapeutic target [26]. In BC patients, high FUT8 protein expression is correlated with lymphatic metastasis and stage status, whereas reduced FUT8 expression is correlated with disease-free survival and overall survival [20].

FUT8 expression level has been linked to tumor clinical features and outcomes in numerous studies. However, few attempts have been made to systematically evaluate such associations. We performed a systematic review and meta-analysis based on collection of references and Gene Expression Omnibus (GEO) microarray data, to clarify the correlations between FUT8 expression and clinical pathology and patient survival in various common types of cancer.

## RESULTS

### Characteristics of studies included in the meta-analysis

A total of 366 articles were found through database mining and manual searches (see M&M); these comprised 152 articles directly related to FUT8, and 214 articles that included microarray data and were indirectly related to FUT8. After screening out duplicate titles and abstracts, 27 full-text articles and 78 datasets remained; of these, 20 articles and 43 datasets were excluded on the basis of criteria described in M&M, finally leaving 7 articles and 35 microarray datasets (involving 6124 patients) for inclusion in the meta-analysis. Characteristics and quality scores of the included studies are summarized in Table 1. Among these, the 35 datasets involved 7 types of malignant tumors and descriptions of 29 types of clinicopathological features related to FUT8, and the 7 articles involved 5 types of malignant tumors and described correlations between FUT8 expression and patient survival.

**Table 1:** Characteristics of studies included in the meta-analysis.

**Tumor source**	**First author and year**	**Country**	**Ethnicity**	**Number of patients**	**Sample Type**	**Method**	**GEO ID**	**Cutoff**	**Survival**	**Follow-up (Months)**	**Quality Score**	**Reference**
Breast Cancer	Lasham 2012	New Zealand	Caucasian	107	tissue	Microarray	gse36771	average	NR	NR	7	[49]
	Chin 2006	America	Caucasian	130	tissue	Microarray	gse69031	average	NR	NR	7	[50]
	Desmedt 2009	Belgium	Caucasian	55	tissue	Microarray	gse16391	average	NR	NR	6	[51]
	EXPO 2005	America	Caucasian	351	tissue	Microarray	gse2109	average	NR	NR	8	R2 platform
	Lu 2008	America	Caucasian	123	tissue	Microarray	gse5460	average	NR	NR	6	[52]
	Concha 2011	Spain	Caucasian	66	tissue	Microarray	gse29431	average	NR	NR	7	R2 platform
	Iwamoto 2011	America	Caucasian	103	tissue	Microarray	gse22093	average	NR	NR	7	[53]
	Yue 2016	China	Asian	189	tissue	IHC		median	OS, DFS	72	7	[20]
Colorectal Cancer	EXPO 2005	America	Caucasian	315	tissue	Microarray	gse2109	average	NR	NR	7	R2 platform
	Laibe 2012	France	Caucasian	130	tissue	Microarray	gse37892	average	NR	NR	7	[54]
	Jorissen 2009	Australia	Caucasian	290	tissue	Microarray	gse14333	average	NR	NR	8	[55]
	Smith 2010	America	Caucasian	232	tissue	Microarray	gse17538	average	NR	NR	7	[56]
	Watanabe 2006	Japan	Asian	84	tissue	Microarray	gse4554	average	NR	NR	5	[57]
	Tsukamoto 2011	Japan	Asian	148	tissue	Microarray	gse21510	average	NR	NR	7	[58]
	Jorissen 2008	Denmark	Caucasian	155	tissue	Microarray	gse13294	average	NR	NR	6	[59]
	Schlicker 2012	United Kingdom	Caucasian	62	tissue	Microarray	gse35896	average	NR	NR	5	[60]
	Barras 2017	Australia	Caucasian	59	tissue	Microarray	gse75316	average	NR	NR	5	[61]
Ependymoma	Donson 2009	America	Caucasian	19	tissue	Microarray	gse16155	average	NR	NR	7	[62]
	Johnson 2010	America	Caucasian	83	tissue	Microarray	NR	average	NR	NR	7	[63]
	Hoffman 2014	America	Caucasian	65	tissue	Microarray	gse50385	average	NR	NR	7	[64]
	Vladoiu 2019	Germany	Caucasian	209	tissue	Microarray	gse64415	average	NR	NR	8	[65]
Glioma	Freije 2004	America	Caucasian	85	tissue	Microarray	gse4412	average	NR	NR	6	[66]
	Gravendeel 2009	Netherlands	Caucasian	276	tissue	Microarray	gse16011	average	OS	240	7	[67]
	Kawaguchi 2013	Japan	Asian	50	tissue	Microarray	gse43378	average	NR	NR	6	[68]
	Zhang 2014	America	Caucasian	21	tissue	Microarray	gse50774	average	NR	NR	6	[69]
Non-Small Cell Cancer	Tarca 2013	Switzerland	Caucasian	150	tissue	Microarray	gse43580	average	NR	NR	7	[70]
	Muley 2014	Germany	Caucasian	100	tissue	Microarray	gse33532	average	NR	NR	8	R2 platform
	Honma 2015	Japan	Asian	129	tissue	IHC		median	OS	168	7	[71]
	Chen 2013	China	Asian	140	tissue	IHC		median	OS, DFS	120	7	[21]
	Wu 2019	China	Asian	135	tissue	IHC		median	OS, DFS	60	7	[72]
	Park 2020	Korea	Asian	217	tissue	Microarray	gse31210	median	DFS	120	8	[73]
Medulloblastoma	Robinson 2012	America	Caucasian	76	tissue	Microarray	gse37418	average	NR	NR	6	[74]
	Northcott 2017	Germany	Caucasian	223	tissue	Microarray	NR	average	NR	NR	7	[75]
	Kool 2008	Netherlands	Caucasian	62	tissue	Microarray	gse10327	average	NR	NR	7	[76]
	Delattre 2012	NR	NR	57	tissue	Microarray	NR	average	NR	NR	6	R2 platform
Neuroblastoma	Delattre 2009	France	Caucasian	34	tissue	Microarray	gse14880	average	NR	NR	5	R2 platform
	Ohtaki 2010	Japan	Asian	51	tissue	Microarray	gse16237	average	NR	NR	6	[77]
	Lastowska 2007	United Kingdom	Caucasian	30	tissue	Microarray	gse13136	average	NR	NR	5	[78]
	Molenaar 2012	Netherlands	Caucasian	88	tissue	Microarray	gse16476	average	NR	NR	7	[79]
Pancreatic Ductal Adenocarcinoma	Tada 2019	Japan	Asian	62	tissue	IHC		median	RFS	120	7	[80]
Diffuse Large B Cell Lymphoma	Xiao 2008	America	Caucasian	420	tissue	Microarray	gse10846	average	OS	240	8	R2 platform
Gastric Cancer	Tan 2018	Switzerland	Caucasian	192	tissue	Microarray	gse15459	average	OS	60	7	R2 platform

OS, overall survival; DFS, disease-free survival; RFS, relapse-free survival; IHC, immunohistochemistry; NR, not reported.

### Association of FUT8 expression with clinicopathological features of various types of malignant tumors

**Figure 1:**
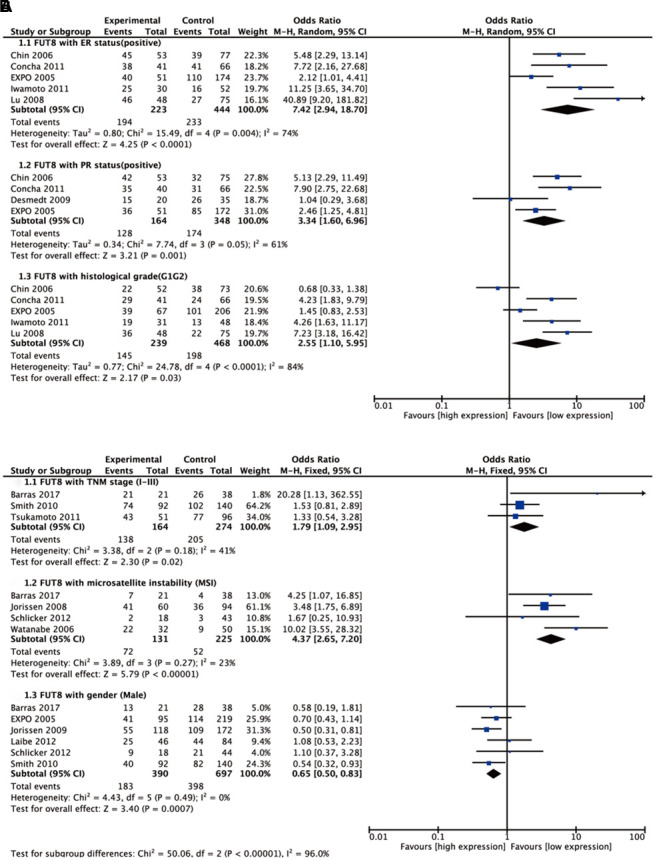
**Forest plots of the significant associations between FUT8 expression and clinical features in two tumor types**. (**A**) breast cancer; (**B**) colorectal cancer.

**Figure 2:**
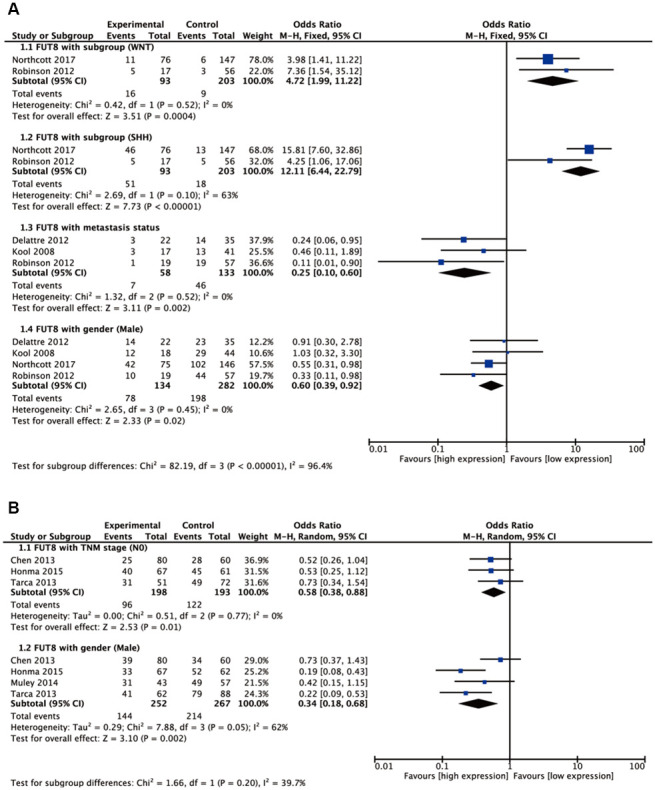
**Forest plots of significant associations between FUT8 expression and clinical features in two tumor types.** (**A**) medulloblastoma; (**B**) non-small cell lung cancer.

Seven malignant tumors (ependymoma, glioma, BC, colorectal cancer (CRC), medulloblastoma (MBL), neuroblastoma (NB), NSCLC) were included in meta-analyses for clinicopathological features. Pooled results are presented in Supplementary Table 1. For BC, high FUT8 expression level was related to positive PR and positive ER status (odds ratio [OR]= 3.34, 95% confidence interval [CI]: 1.60-6.96, p= 0.001 and odds ratio [OR]= 7.42, 95% confidence interval [CI]: 2.94-18.7, p< 0.0001). FUT8 expression level was also correlated with tumor histological grade (OR= 2.55, 95% CI: 1.10-5.95, p= 0.03) (Figure 1A). For CRC, elevated FUT8 expression level was associated with TNM stage I-III (OR= 1.79, 95% CI: 1.09–2.95, p= 0.02), and was also related to microsatellite instability (MSI) (OR= 4.37, 95% CI: 2.65-7.20, p< 0.00001) and female gender (OR= 0.65, 95% CI: 0.50-0.83, p= 0.0007) (Figure 1B). For ependymoma, high FUT8 expression level was related to patient age (≤10 years) (OR= 3.69, 95% CI: 2.28-5.99, p< 0.00001) and tumor recurrence status (OR= 2.29, 95% CI: 1.07-4.93, p= 0.03) (Supplementary Figure 1A). For glioma, increased FUT8 level was associated with glioblastoma multiforme (GBM) (OR= 1.69, 95% CI: 1.07–2.66, p= 0.02), and FUT8 level was inversely correlated with patient age (≤40 years) (OR= 0.58, 95% CI: 0.35-0.95, p= 0.03) (Supplementary Figure 1B). For MBL, high FUT8 expression level was related to Wingless (WNT) (OR= 4.72, 95% CI: 1.99-11.22, p= 0.0004) and Sonic Hedgehog (SHH) molecular subgroups (OR= 12.11, 95% CI: 6.44-22.79, p< 0.00001). FUT8 level was inversely correlated with metastasis status (OR= 0.25, 95% CI: 0.10-0.60, p= 0.002) and male gender (OR= 0.60, 95% CI: 0.39-0.92, p= 0.02) (Figure 2A). For NSCLC, FUT8 expression level was inversely correlated with N0 status of TNM stage (OR= 0.58, 95% CI: 0.38-0.88, p= 0.01) and male gender (OR= 0.34, 95% CI: 0.18-0.68, p= 0.002) (Figure 2B). Clinicopathological features of malignant tumors related to FUT8 are summarized in Table 2. High FUT8 expression level in NB showed no association with any clinicopathological feature, including INSS stage, gender, or mycn amplified status (Supplementary Figure 2).

**Table 2:** Clinicopathological features related to enhanced FUT8 expression level in various malignant tumors.

**Tumor source**	**Clinicopathological features**	**Reference**
Breast cancer	ER status positive, PR status positive, histological grade (G1G2) histological grade (G1)	[50], [51], [52], [53], R2 platform
Colorectal cancer	Duke stage A, Duke stage AB, location (Left), gender (male) microsatellite instability (MSI)	[54], [55], [56], [57], [58], [59], [60], [61], R2 platform
Glioma	Age (≤40 years), glioblastoma multiforme (GBM)	[66], [67], [68], [69]
Medulloblastoma	Molecular subgroups (G3), molecular subgroups (G4), molecular subgroups (SHH), molecular subgroups (WNT) Gender (male), metastasis	[74], [75], [76], R2 platform
Non-small cell lung cancer	Gender (male), TNM stage N0, TNM stage M1	[21], [70], [71], R2 platform
Ependymoma	Age (≤10 years), recurrence	[63], [64], [65]

ER, estrogen receptor; PR, progesterone receptor.

### Prognostic value of FUT8 expression in survival of various types of malignant tumors

**Figure 3:**
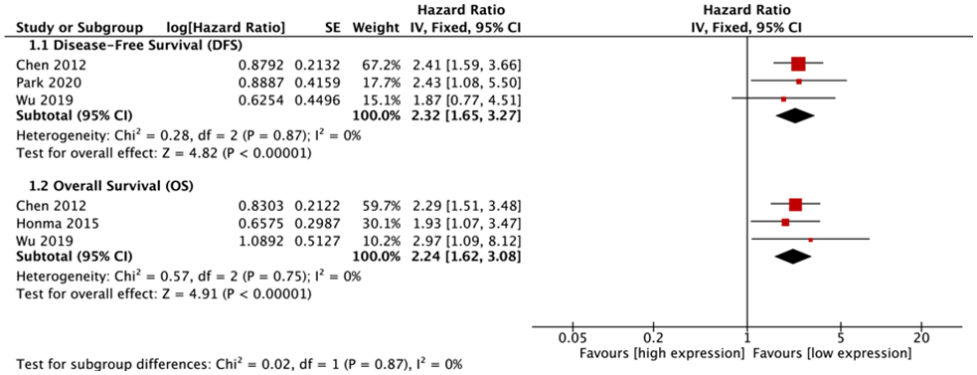
**Forest plots of associations between FUT8 expression and non-small cell lung cancer overall survival.**

Five types of malignant tumors (NSCLC, glioma, diffuse large B cell lymphoma (DLBCL), gastric cancer (GC), BC) were included in meta-analyses for survival. For NSCLC, elevated FUT8 expression level was associated with shorter disease-free survival (DFS) (hazard ratio [HR]= 2.32, 95% CI: 1.65-3.27, p< 0.00001), and with lower overall survival (OS) (HR= 2.24, 95% CI: 1.62-3.08, p< 0.00001) (Figure 3).

**Figure 4:**
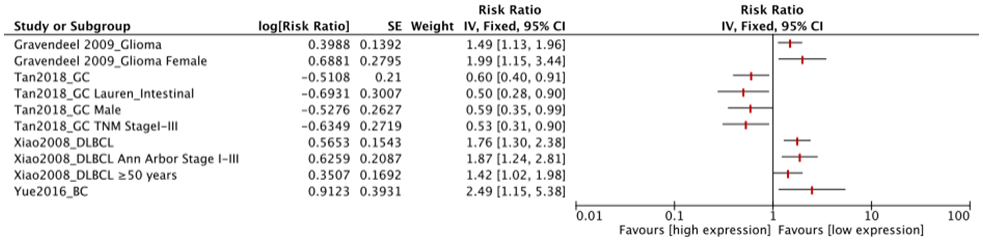
**Forest plots of associations between FUT8 expression and tumor overall survival in single clinical features studied.**

FUT8 expression levels in the other 4 tumor types (glioma, DLBCL, GC, BC) were also correlated with OS (Figure 4). Among these, high FUT8 expression was associated with shorter OS in glioma (RR= 1.49, 95% CI: 1.13-1.96), DLBCL (RR= 1.76, 95% CI: 1.30-2.38), and BC (RR= 2.49, 95% CI: 1.15-5.38), but with longer OS in GC (RR= 0.60, 95% CI: 0.40-0.91). For glioma, upregulated FUT8 expression was associated with shorter OS in female patients (RR= 1.99, 95% CI: 1.15-3.44). For DLBCL, high FUT8 expression was associated with shorter OS in Ann Arbor stages I-III (RR= 1.87, 95% CI: 1.24-2.81) and in patients aged >50 years (RR= 1.42, 95% CI: 1.02-1.98). For GC, high FUT8 expression was associated with better OS in intestinal-type Lauren classification (RR= 0.50, 95% CI: 0.28-0.90), males (RR= 0.59, 95% CI: 0.35-0.99), and TNM stage I-III patients (RR= 0.53, 95% CI: 0.31-0.90). Pooled survival results are presented in Supplementary Table 2.

## DISCUSSION

FUT8 is clearly involved in tumor initiation and progression, and in various biological behaviors of cancer, including cell proliferation, apoptosis, migration, and metastasis [27]. Prognostic values of glycosylation or fucosylated antigens in many types of cancer have been documented in several previous reviews and meta-analyses [14, 15, 28–30]. However, very little meta-analysis of FUT8 has been performed, because of lack of sufficient studies and references. Many reports are based on use of microarrays to study cancer genomes; accordingly, we performed a systematic review after collecting GEO microarray data, and data from a large number of studies, focused on the prognostic value of FUT8 in cancer. This review / meta-analysis is, to our knowledge, the first to comprehensively clarify the association of FUT8 expression with specific clinicopathological features and survival data for various types of cancer.

Our analyses demonstrated that FUT8 expression levels were most often associated with tumor stage (n=4: BC, CRC, MBL, NSCLC), molecular classification (n=2: BC, MBL), age (n=2: glioma, ependymoma), and gender (n=2: MBL, NSCLC), but less associated with histological grade (n=1: BC) and pathological typing (n=1: glioma). FUT8 expression showed no association with disease induction, location, or family history for any of the above cancer types. These findings suggest that FUT8 plays an intrinsic role mainly in tumor development, and is therefore a potentially important biomarker for malignant tumors. Previous studies have focused mainly on the relationship between FUT8 expression and OS for survival. The majority of such studies (n=5: NSCLC, BC, DLBCL, GC, glioma) found significant correlations of FUT8 expression with OS of tumor, and also associations between survival and clinicopathological features, particularly tumor stage. A few studies focused on associations of FUT8 expression with other types of survival (DFS, RFS). For the research on pathways related to FUT8 and tumor survival, many references indicate that FUT8 activates several related signaling pathways, including Ras/MAPKK signaling, c-Met signaling, Akt/mTOR signaling and Wnt/β-catenin signaling, which ultimately lead to hepatocellular carcinoma or colorectal cancer affects the overall survival of patients [31–33]. Höti et al demonstrated that overexpression of FUT8 resulted in upregulation of epidermal growth factor receptor (EGFR) and corresponding downstream signaling, leading to increased prostate cancer cells survival [34]. In short, findings of these studies, taken together, demonstrate the strong prognostic value of FUT8 expression in malignant tumors.

NSCLC is the tumor type most frequently studied in regard to FUT8 expression status. High FUT8 expression was inversely correlated with N0 status of TNM stage and with male gender. High FUT8 expression was associated with shorter DFS, and with lower OS. Thus, FUT8 should be a useful survival prognostic predictor for female NSCLC patients with high FUT8 expression. Our analyses revealed an association of high FUT8 expression level with positive ER and PR status, and with shorter OS, in BC patients. FUT8 expression is therefore presumably enhanced in luminal A and B BC patients, and may be a useful survival prognostic predictor in such patients. A 2019 study indicated that FUT8 is highly expressed in ER+ BC patients, is associated with metastasis, and is a potential therapeutic target in these patients [35].

Microsatellite instability (MSI) is a molecular "fingerprint" of defective mismatch repair systems, and methods to detect MSI are well established and routinely incorporated into clinical practice. Prognosis for MSI tumors is better than that for microsatellite stable CRC [36]. In regard to CRC, we found that high FUT8 expression was associated with MSI, female gender, and TNM stages I-III. E-cadherin, a Ca^2+^-dependent cell adhesion molecule, was found to be significantly enhanced in dense culture of FUT8-transfected colorectal adenocarcinoma cells, resulting in increased cell-cell adhesion [23]. In addition, E-cadherin truncation was significantly higher in low MSI as compared with high MSI tumors [37]. The inferred mechanism is that the low expression of FUT8 in TNM stages IV or microsatellite stable colorectal cancer may cause E-cadherin to decrease or be truncated, resulting in decreased tumor cell-cell adhesion and increased metastasis.

Medulloblastoma, a common type of malignant brain cancer that accounts for 8-10% of childhood brain tumors [38], comprises four molecular subgroups (WNT, SHH, group 3, group 4) that have differing prognoses [39]. In general, subgroup WNT has very good prognosis and rare metastasis; subgroup SHH has good prognosis in infants and intermediate prognosis and uncommon metastasis in older children; subgroup G4 has intermediate prognosis and frequent metastasis; subgroup G3 has poor prognosis and very frequent metastasis [40–43]. Our analysis revealed association of high FUT8 expression with subgroups WNT and SHH, and inverse correlation with metastasis and male gender. High FUT8 expression in medulloblastoma therefore appears to be a positive prognostic indicator, particularly for female patients. In regard to NB, we did not find any notable relationship of high FUT8 expression with clinicopathological features. On the other hand, another group reported aberrant expression of N-glycans and short O-glycans in NB cells, and regulation of their expression levels by associated glycosyltransferases [44]. GnT-V expression in NB patients was correlated with favorable prognosis and treatment outcome [45]. GALNT9 was expressed in neuroblasts derived from primary tumor [46] but not in those derived from metastatic bone marrow, and may be a useful prognostic marker for positive clinical outcome in NB patients [47].

Overall, our results are comprehensive and seemingly reliable in view of the high quality of included articles and microarray data. On the other hand, there are inherent limitations in our analysis. (i) Heterogeneity is present among studies of a given tumor type, and is difficult to address because of methodological differences (*e.g.*, sample selection, detection method, determination of cutoff value, statistical analysis). (ii) Nearly all our included studies report a statistically significant result. Although a Begg’s funnel plot indicates absence of publication bias (Supplementary Figure 3), our experience suggests that selective reporting bias is common in the literature regarding FUT8 and tumor prognosis. (iii) Roughly half of our included studies had small sample size (<100), which is often associated with inflated estimates of effect size and with high heterogeneity.

Results of our analyses – with due consideration of the above caveats – highlight the prognostic value of FUT8 expression in a variety of malignant tumors, and the important biological function of FUT8 in tumor progression. FUT8 may exert its effects in such tumors by regulating functional protein core fucosylations involved in tumor development and metastasis (*e.g.*, L1CAM, P53, TGF-β, EGFR) [25, 34, 48]. Thus, dysregulation of FUT8 would have effects on tumor development, and consequently on clinical prognosis of patients. The molecular mechanisms remain to be clarified, and are being addressed in ongoing studies.

In conclusion, our systematic review and meta-analysis confirmed the association of FUT8 with clinicopathological features and patient survival rates for certain malignant tumor types. FUT8 expression is a significant predictor of prognosis for these tumors. The relative weight of FUT8 correlations with particular clinical features is currently difficult to evaluate because of the presence of many uncontrollable factors. Reliable verification of prognostic value of FUT8 in these tumor types will require a large-scale study using standardized methods of detection, analysis, and reporting.

## MATERIALS AND METHODS

We performed this review based on PRISMA (Preferred Reporting Items for Systematic Reviews and Meta-Analyses) criteria.

### Search strategy

The PubMed, Embase, Web of Science, Springer, Chinese National Knowledge Infrastructure (CNKI), and Wan Fang databases were searched systematically to identify and retrieve all pertinent publications through June 24, 2020. Keywords and search terms used were: FUT8 or Fut8 or Fucosyltransferase 8 or α1-6 Fucosyltransferase or Core fucosylation or Fucosyltransferase; Malignant tumor or Cancer or Carcinoma or Neoplasm; and clinical or clinicopathological or clinicopathology or prognosis predictor or survival or Odds Ratio (OR) or Hazard Ratio (HR). References in retrieved articles were screened manually. Languages of retrieved articles were restricted to English and Chinese.

### Data extraction and analysis of GEO datasets

Our first step was to read and screen articles related to tumor clinicopathological features and prognosis, and search for microarray datasets through the references. In this way, 96 datasets from 214 articles were extracted for preliminary screening. The second step was to re-screen the 96 datasets in regard to presence of FUT8 data, an appropriate clinical pathology track, and sufficient number of samples. In this way, 35 malignant tumor microarray datasets were selected and downloaded from the Gene Expression Omnibus (GEO) database (https://www.ncbi.nlm.nih.gov/gds) and R2 platform (http://r2.amc.nl). These datasets included a total of 4918 samples, from 7 types of malignant tumor: breast cancer (BC), colorectal cancer (CRC), ependymoma, glioma, non-small cell lung cancer (NSCLC), medulloblastoma (MBL), and neuroblastoma (NB). The datasets all used the Affymetrix Human Genome U133a or U133 Plus 2.0 expression arrays to detect expression value signals. Data were normalized using Microarray Suite (MAS) V. 5.0. In many cases, more than one probeset has been reported for FUT8 gene. We selected a probeset with highest average presence signal (APS) by default. mRNA expression of FUT8 was assigned to "low" or "high" category based on average expression value of each dataset.

### Inclusion and exclusion criteria

**Figure 5:**
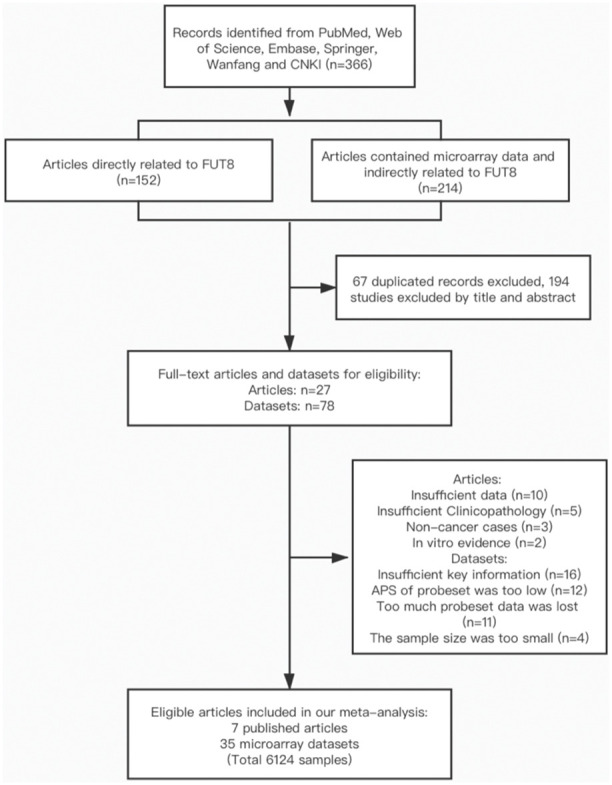
**Flow diagram of article selection process.**

Studies were included if they met the following criteria: (1) original study focused on human subjects; (2) presented FUT8 expression data and malignant tumor clinicopathological features and/or survival data; (3) reported an OR or HR with 95% confidence interval (CI), or sufficient data were presented so that we could calculate them; (4) full text was available. Exclusion criteria were as follows: (1) study lacked key information (*e.g.*, clinical parameters, survival curves), or usable data; (2) APS of probeset was too low, or too many data were lost; (3) HRs applied to a combination of multiple FUTs; (4) article was a review, letter, single case report, or conference abstracts. In cases of multiple articles from the same group, reporting overlapping data, only the most complete one was included. The article selection process is shown in flow diagram form in Figure 5.

### Quality assessment and data extraction

Two authors (MXM and GXH) reviewed potentially eligible articles independently. Quality of each study was assessed using the Newcastle-Ottawa Scale. The following information was extracted from each included study:

(1) basic information: first author’s or uploaded dataset author’s name, publication year, country of origin, names of malignant tumors, sample size, FUT8 expression levels, detection methods, sample type, outcome measurements, follow-up duration, cutoff value, survival analysis method; (2) p values for correlation between FUT8 expression and clinicopathological features of malignant tumors, and original data used for calculation of ORs and their 95% CIs; (3) HRs and their 95% CIs for survival analysis. If HRs were not directly accessible in the text, Kaplan-Meier survival curves were read using Engauge Digitizer (V. 4.1) to obtain data. Different datasets for a particular malignant tumor were considered as separate studies, and respective HRs were extracted. In cases of possible discrepancy, a consensus was reached by discussion among all authors.

### Statistical analysis

ORs and their 95% CIs were used to estimate associations of FUT8 with clinical features of malignant tumors. For purposes of comparison, patients were divided into paired categories (*e.g.*, male vs. female; TNM stages I, II and III vs. IV; ER/PR status positive vs. negative). For survival rates, HRs with corresponding 95% CIs were used. All ORs and HRs were calculated for high FUT8 expression. When a given FUT8 was investigated in two or more different studies, a meta-analysis was performed to combine the effect size. Z test was used to determine significance of ORs or HRs. Heterogeneity between studies was tested using Q statistic and I^2^ test. When I^2^ value was >50% (indicating significant heterogeneity) a random-effects model was used. For I^2^ value ≤50%, a fixed-effects model was used. Statistical analyses were performed with software program Review Manager V. 5.3 (Cochrane Collaboration; London, UK). Differences with p< 0.05 were considered statistically significant.

## Supplementary Material

Supplementary Figures

Supplementary Table 1

Supplementary Table 2
