# Supplementary material for: Role of FUT8 expression in clinicopathology and patient survival for various malignant tumor types: a systematic review and meta-analysis
Source: Aging (Albany NY). 2020 Dec 11;13(2):2212–30. doi: 10.18632/aging.202239 (PMC7880376; doi:10.18632/aging.202239)
Supplement: Supplementary Figures [file aging-13-202239-s001.pdf]

SUPPLEMENTARY FIGURES

A

Study or Subgroup	Experimental Events	Experimental Total	Control Events	Control Total	Weight	Odds Ratio M-H, Fixed, 95% CI
**1.1 FUT8 with patient's age (≤10 years)**						
Johnson 2010	24	37	28	98	31.7%	4.62 [2.06, 10.32]
Vladoiu 2019	53	82	37	103	68.3%	3.26 [1.78, 5.97]
**Subtotal (95% CI)**		**119**		**201**	**100.0%**	**3.69 [2.28, 5.99]**
Total events	77		65			
Heterogeneity: $Chi^2 = 0.46$, df = 1 (P = 0.50); $I^2 = 0\%$						
Test for overall effect: Z = 5.29 (P < 0.00001)						
**1.2 FUT8 with tumor recurrence**						
Hoffman 2014	13	31	7	34	44.3%	2.79 [0.93, 8.33]
Vladoiu 2019	9	87	6	105	55.7%	1.90 [0.65, 5.58]
**Subtotal (95% CI)**		**118**		**139**	**100.0%**	**2.29 [1.07, 4.93]**
Total events	22		13			
Heterogeneity: $Chi^2 = 0.24$, df = 1 (P = 0.63); $I^2 = 0\%$						
Test for overall effect: Z = 2.13 (P = 0.03)						

Test for subgroup differences: $Chi^2 = 1.06$, df = 1 (P = 0.30), $I^2 = 5.4\%$

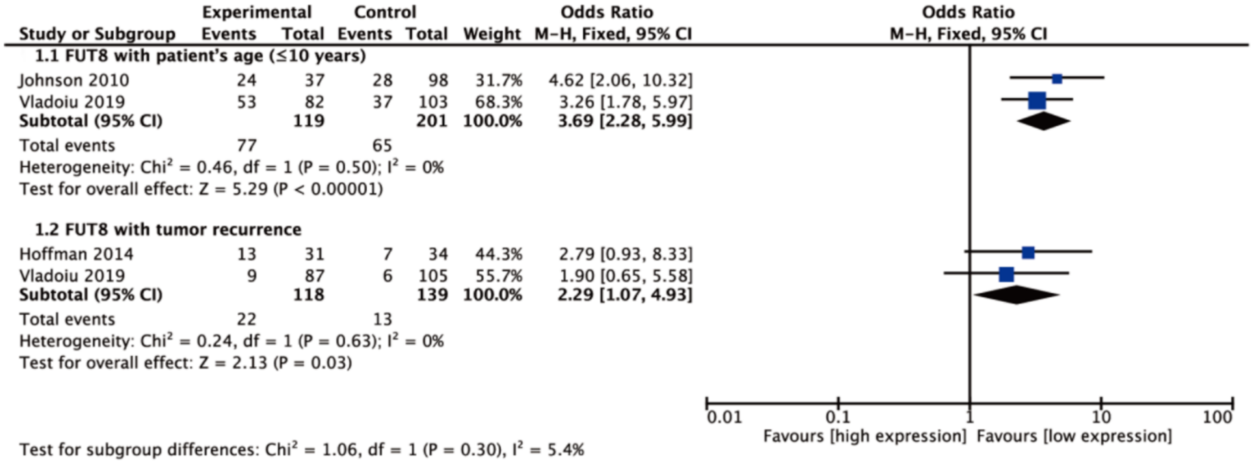

B

Study or Subgroup	Experimental Events	Experimental Total	Control Events	Control Total	Weight	Odds Ratio M-H, Fixed, 95% CI
**1.1 FUT8 with patient's age (≤40 years)**						
Gravendeel 2009	25	110	54	166	79.0%	0.61 [0.35, 1.06]
Kawaguchi 2013	2	17	10	33	14.2%	0.31 [0.06, 1.60]
Zhang 2014	4	9	6	12	6.8%	0.80 [0.14, 4.53]
**Subtotal (95% CI)**		**136**		**211**	**100.0%**	**0.58 [0.35, 0.95]**
Total events	31		70			
Heterogeneity: $Chi^2 = 0.74$, df = 2 (P = 0.69); $I^2 = 0\%$						
Test for overall effect: Z = 2.15 (P = 0.03)						
**1.2 FUT8 with glioblastoma multiforme (GBM)**						
Freije 2004	27	30	44	55	10.7%	2.25 [0.58, 8.80]
Gravendeel 2009	73	116	86	168	89.3%	1.62 [1.00, 2.62]
**Subtotal (95% CI)**		**146**		**223**	**100.0%**	**1.69 [1.07, 2.66]**
Total events	100		130			
Heterogeneity: $Chi^2 = 0.20$, df = 1 (P = 0.66); $I^2 = 0\%$						
Test for overall effect: Z = 2.25 (P = 0.02)						

Test for subgroup differences: $Chi^2 = 9.64$, df = 1 (P = 0.002), $I^2 = 89.6\%$

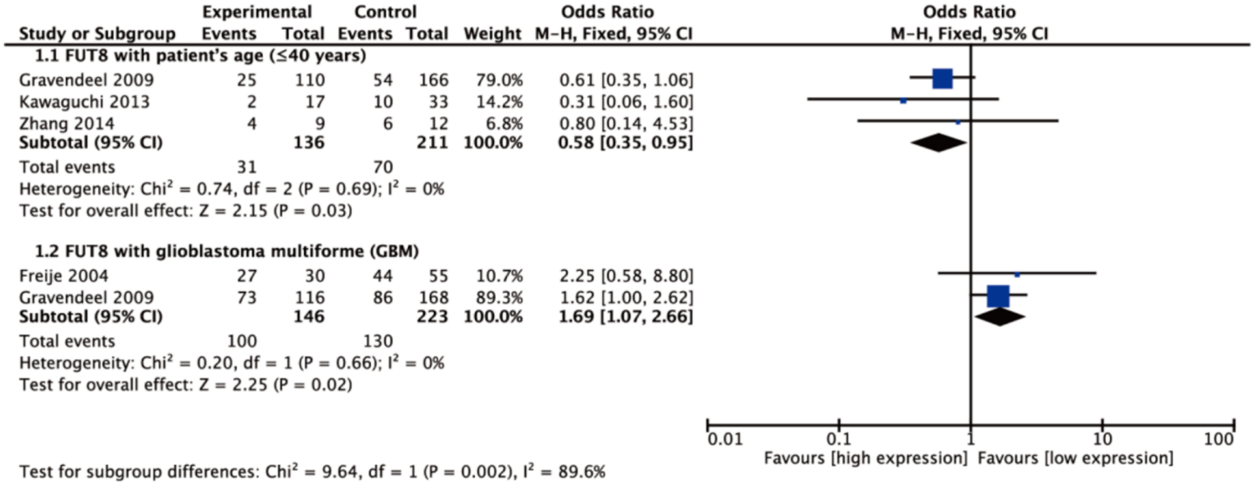

Supplementary Figure 1. Forest plots of the significant associations between FUT8 expression and clinical features in two tumors. (**A**, Ependymoma; **B**, Glioma). Footnote as for Figure 1.

Study or Subgroup	Experimental Events	Experimental Total	Control Events	Control Total	Weight	Odds Ratio M-H, Fixed, 95% CI
**1.1 FUT8 with Male**						
Lastowska 2007	7	13	13	17	5.4%	0.36 [0.08, 1.71]
Molenaar 2012	22	36	31	52	10.2%	1.06 [0.45, 2.54]
**Subtotal (95% CI)**		**49**		**69**	**15.6%**	**0.82 [0.39, 1.74]**
Total events	29		44			
Heterogeneity: $Chi^2 = 1.42$, df = 1 (P = 0.23); $I^2 = 29\%$						
Test for overall effect: Z = 0.52 (P = 0.61)						
**1.2 FUT8 with mycn_amplify**						
Delattre 2009	4	15	8	15	6.1%	0.32 [0.07, 1.47]
Lastowska 2007	5	13	5	17	2.8%	1.50 [0.33, 6.92]
Molenaar 2012	5	36	11	52	8.0%	0.60 [0.19, 1.91]
Ohtaki 2010	2	16	5	35	2.8%	0.86 [0.15, 4.97]
**Subtotal (95% CI)**		**80**		**119**	**19.7%**	**0.68 [0.33, 1.38]**
Total events	16		29			
Heterogeneity: $Chi^2 = 2.09$, df = 3 (P = 0.55); $I^2 = 0\%$						
Test for overall effect: Z = 1.08 (P = 0.28)						
**1.3 FUT8 with INSS stage 1**						
Delattre 2009	4	15	3	18	2.1%	1.82 [0.34, 9.82]
Molenaar 2012	3	36	5	52	3.9%	0.85 [0.19, 3.83]
Ohtaki 2010	4	16	17	35	8.3%	0.35 [0.10, 1.31]
**Subtotal (95% CI)**		**67**		**105**	**14.2%**	**0.70 [0.31, 1.60]**
Total events	11		25			
Heterogeneity: $Chi^2 = 2.34$, df = 2 (P = 0.31); $I^2 = 15\%$						
Test for overall effect: Z = 0.84 (P = 0.40)						
**1.4 FUT8 with INSS stage 1-2**						
Delattre 2009	8	15	7	18	3.1%	1.80 [0.45, 7.20]
Molenaar 2012	10	36	13	52	8.0%	1.15 [0.44, 3.02]
Ohtaki 2010	8	16	19	35	6.2%	0.84 [0.26, 2.75]
**Subtotal (95% CI)**		**67**		**105**	**17.2%**	**1.16 [0.60, 2.23]**
Total events	26		39			
Heterogeneity: $Chi^2 = 0.66$, df = 2 (P = 0.72); $I^2 = 0\%$						
Test for overall effect: Z = 0.44 (P = 0.66)						
**1.5 FUT8 with INSS stage 2-3**						
Delattre 2009	6	15	7	18	4.0%	1.05 [0.26, 4.26]
Molenaar 2012	12	36	16	52	9.0%	1.13 [0.45, 2.79]
Ohtaki 2010	6	16	5	35	2.0%	3.60 [0.90, 14.39]
**Subtotal (95% CI)**		**67**		**105**	**15.0%**	**1.44 [0.74, 2.79]**
Total events	24		28			
Heterogeneity: $Chi^2 = 2.16$, df = 2 (P = 0.34); $I^2 = 7\%$						
Test for overall effect: Z = 1.08 (P = 0.28)						
**1.6 FUT8 with INSS stage 1-3**						
Delattre 2009	11	15	10	18	2.5%	2.20 [0.50, 9.61]
Molenaar 2012	15	36	21	52	10.4%	1.05 [0.44, 2.50]
Ohtaki 2010	10	16	22	35	5.4%	0.98 [0.29, 3.34]
**Subtotal (95% CI)**		**67**		**105**	**18.2%**	**1.19 [0.63, 2.24]**
Total events	36		53			
Heterogeneity: $Chi^2 = 0.83$, df = 2 (P = 0.66); $I^2 = 0\%$						
Test for overall effect: Z = 0.54 (P = 0.59)						
**Total (95% CI)**		**397**		**608**	**100.0%**	**0.99 [0.75, 1.32]**
Total events	142		218			
Heterogeneity: $Chi^2 = 13.13$, df = 17 (P = 0.73); $I^2 = 0\%$						
Test for overall effect: Z = 0.04 (P = 0.97)						
Test for subgroup differences: $Chi^2 = 3.78$, df = 5 (P = 0.58), $I^2 = 0\%$						

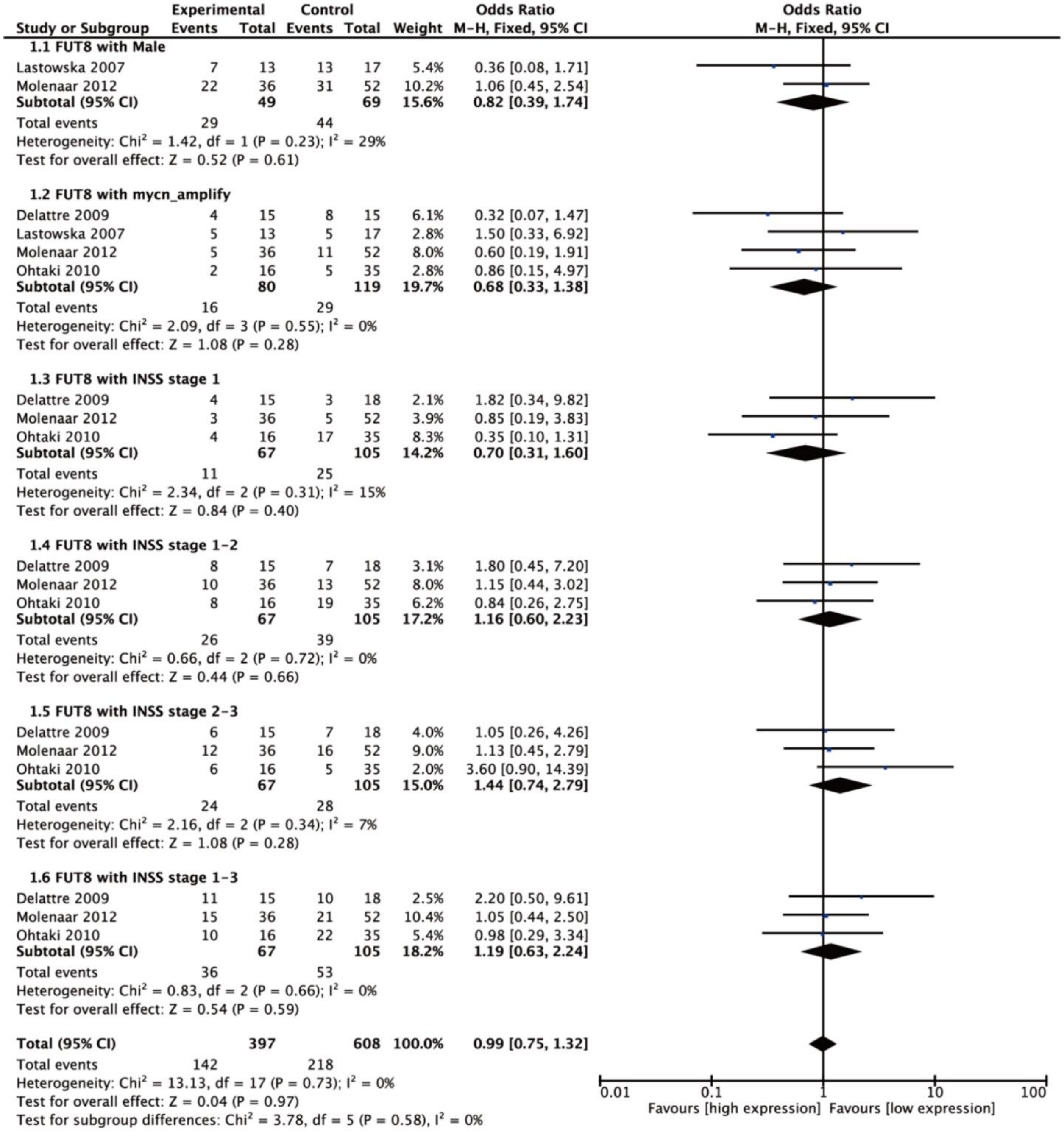

Supplementary Figure 2. Forest plots of the significant associations between FUT8 expression and clinical features in neuroblastoma. Footnote as for Figure. 1.

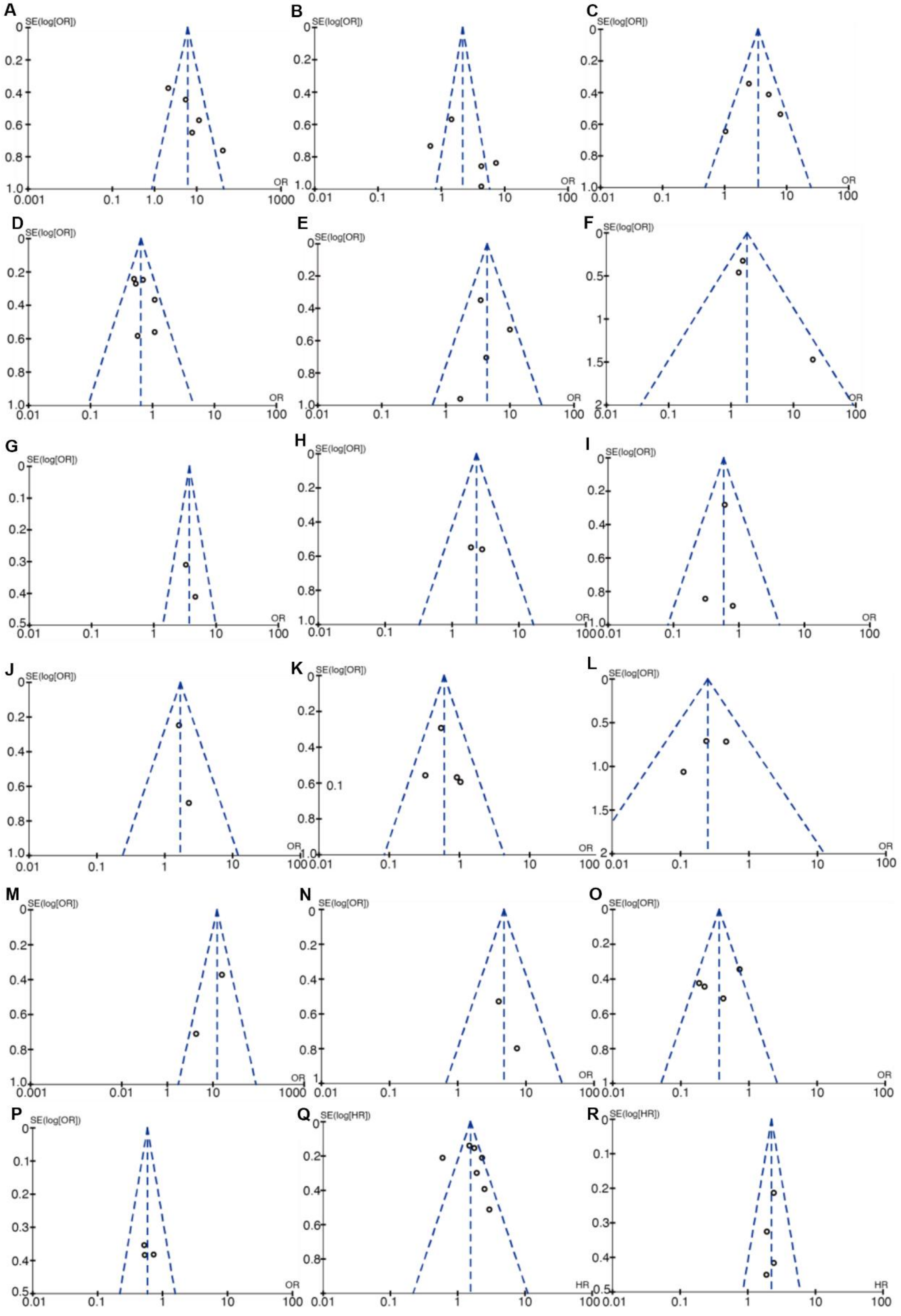

Supplementary Figure 3. Begg's funnel plot for publication bias on the associations between the expression of FUT8 and survival analysis & tumor clinical features. Each point represents a single study for the indicated association. (**A**) ER status in BC; (**B**) histological grade in BC; (**C**) PR status in BC; (**D**) gender in CRC; (**E**) MSI in CRC; (**F**) TNM stage in CRC; (**G**) age in ependymoma; (**H**) tumor recurrence in ependymoma; (**I**) age in glioma; (**J**) GBM in glioma; (**K**) gender in MBL; (**L**) metastasis in MBL; (**M**) SHH in MBL; (**N**) WNT in MBL; (**O**) gender in NSCLC; (**P**) TNM stage in NSCLC; (**Q**) OS in tumors ; (**R**) DFS in tumors.
