# Supplementary material for: Role of FUT8 expression in clinicopathology and patient survival for various malignant tumor types: a systematic review and meta-analysis
Source: Aging (Albany NY). 2020 Dec 11;13(2):2212–30. doi: 10.18632/aging.202239 (PMC7880376; doi:10.18632/aging.202239)
Supplement: Supplementary Table 1 [file aging-13-202239-s002.docx]

| Tumor source | First author & year | Country | Ethnicity | Number of patients | Sample Type | Method | GEO ID | Cutoff | Survival | Follow-up (Months) | Quality Score | Reference |
| --- | --- | --- | --- | --- | --- | --- | --- | --- | --- | --- | --- | --- |
| Breast Cancer | Lasham 2012 | New Zealand | Caucasian | 107 | tissue | Microarray | gse36771 | average | NR | NR | 7 | [43] |
|  | Chin 2006 | America | Caucasian | 130 | tissue | Microarray | gse69031 | average | NR | NR | 7 | [44] |
|  | Desmedt 2009 | Belgium | Caucasian | 55 | tissue | Microarray | gse16391 | average | NR | NR | 6 | [45] |
|  | EXPO 2005 | America | Caucasian | 351 | tissue | Microarray | gse2109 | average | NR | NR | 8 | R2 platform |
|  | Lu 2008 | America | Caucasian | 123 | tissue | Microarray | gse5460 | average | NR | NR | 6 | [46] |
|  | Concha 2011 | Spain | Caucasian | 66 | tissue | Microarray | gse29431 | average | NR | NR | 7 | R2 platform |
|  | Iwamoto 2011 | America | Caucasian | 103 | tissue | Microarray | gse22093 | average | NR | NR | 7 | [47] |
|  | Yue 2016 | China | Asian | 189 | tissue | IHC |  | median | OS, DFS | 72 | 7 | [20] |
| Colorectal Cancer | EXPO 2005 | America | Caucasian | 315 | tissue | Microarray | gse2109 | average | NR | NR | 7 | R2 platform |
|  | Laibe 2012 | France | Caucasian | 130 | tissue | Microarray | gse37892 | average | NR | NR | 7 | [48] |
|  | Jorissen 2009 | Australia | Caucasian | 290 | tissue | Microarray | gse14333 | average | NR | NR | 8 | [49] |
|  | Smith 2010 | America | Caucasian | 232 | tissue | Microarray | gse17538 | average | NR | NR | 7 | [50] |
|  | Watanabe 2006 | Japan | Asian | 84 | tissue | Microarray | gse4554 | average | NR | NR | 5 | [51] |
|  | Tsukamoto 2011 | Japan | Asian | 148 | tissue | Microarray | gse21510 | average | NR | NR | 7 | [52] |
|  | Jorissen 2008 | Denmark | Caucasian | 155 | tissue | Microarray | gse13294 | average | NR | NR | 6 | [53] |
|  | Schlicker 2012 | United Kingdom | Caucasian | 62 | tissue | Microarray | gse35896 | average | NR | NR | 5 | [54] |
|  | Barras 2017 | Australia | Caucasian | 59 | tissue | Microarray | gse75316 | average | NR | NR | 5 | [55] |
| Ependymoma | Donson 2009 | America | Caucasian | 19 | tissue | Microarray | gse16155 | average | NR | NR | 7 | [56] |
|  | Johnson 2010 | America | Caucasian | 83 | tissue | Microarray | NR | average | NR | NR | 7 | [57] |
|  | Hoffman 2014 | America | Caucasian | 65 | tissue | Microarray | gse50385 | average | NR | NR | 7 | [58] |
|  | Vladoiu 2019 | Germany | Caucasian | 209 | tissue | Microarray | gse64415 | average | NR | NR | 8 | [59] |
| Glioma | Freije 2004 | America | Caucasian | 85 | tissue | Microarray | gse4412 | average | NR | NR | 6 | [60] |
|  | Gravendeel 2009 | Netherlands | Caucasian | 276 | tissue | Microarray | gse16011 | average | OS | 240 | 7 | [61] |
|  | Kawaguchi 2013 | Japan | Asian | 50 | tissue | Microarray | gse43378 | average | NR | NR | 6 | [62] |
|  | Zhang 2014 | America | Caucasian | 21 | tissue | Microarray | gse50774 | average | NR | NR | 6 | [63] |
| Non-Small Cell Cancer | Tarca 2013 | Switzerland | Caucasian | 150 | tissue | Microarray | gse43580 | average | NR | NR | 7 | [64] |
|  | Muley 2014 | Germany | Caucasian | 100 | tissue | Microarray | gse33532 | average | NR | NR | 8 | R2 platform |
|  | Honma 2015 | Japan | Asian | 129 | tissue | IHC |  | median | OS | 168 | 7 | [65] |
|  | Chen 2013 | China | Asian | 140 | tissue | IHC |  | median | OS, DFS | 120 | 7 | [21] |
|  | Wu 2019 | China | Asian | 135 | tissue | IHC |  | median | OS, DFS | 60 | 7 | [66] |
|  | Park 2020 | Korea | Asian | 217 | tissue | Microarray | gse31210 | median | DFS | 120 | 8 | [67] |
| Medulloblastoma | Robinson 2012 | America | Caucasian | 76 | tissue | Microarray | gse37418 | average | NR | NR | 6 | [68] |
|  | Northcott 2017 | Germany | Caucasian | 223 | tissue | Microarray | NR | average | NR | NR | 7 | [69] |
|  | Kool 2008 | Netherlands | Caucasian | 62 | tissue | Microarray | gse10327 | average | NR | NR | 7 | [70] |
|  | Delattre 2012 | NR | NR | 57 | tissue | Microarray | NR | average | NR | NR | 6 | R2 platform |
| Neuroblastoma | Delattre 2009 | France | Caucasian | 34 | tissue | Microarray | gse14880 | average | NR | NR | 5 | R2 platform |
|  | Ohtaki 2010 | Japan | Asian | 51 | tissue | Microarray | gse16237 | average | NR | NR | 6 | [71] |
|  | Lastowska 2007 | United Kingdom | Caucasian | 30 | tissue | Microarray | gse13136 | average | NR | NR | 5 | [72] |
|  | Molenaar 2012 | Netherlands | Caucasian | 88 | tissue | Microarray | gse16476 | average | NR | NR | 7 | [73] |
| Pancreatic Ductal Adenocarcinoma | Tada 2019 | Japan | Asian | 62 | tissue | IHC |  | median | RFS | 120 | 7 | [74] |
| Diffuse Large B Cell Lymphoma | Xiao 2008 | America | Caucasian | 420 | tissue | Microarray | gse10846 | average | OS | 240 | 8 | R2 platform |
| Gastric Cancer | Tan 2018 | Switzerland | Caucasian | 192 | tissue | Microarray | gse15459 | average | OS | 60 | 7 | R2 platform |

**Supplemeentary Table 1.** Characteristics of studies included in the meta-analysis.

OS, overall survival; DFS, disease-free survival; RFS, relapse-free survival; IHC, immunohistochemistry; NR, not reported.
