# Supplementary material for: Role of FUT8 expression in clinicopathology and patient survival for various malignant tumor types: a systematic review and meta-analysis
Source: Aging (Albany NY). 2020 Dec 11;13(2):2212–30. doi: 10.18632/aging.202239 (PMC7880376; doi:10.18632/aging.202239)
Supplement: Supplementary Table 2 [file aging-13-202239-s003.pdf]

Supplementary Table 2. Meta-analysis results of the associations between the expression of FUT8 and survival of tumors.

Tumor Source	Reference	Subgroup	Survival	HR & 95% CI	Analysis
Non-Small Cell Lung Cancer	Honma 2015		OS	1.93 (1.07-3.47)	Univariate
			OS	1.81 (1.01-3.25)	Multivariate
		TNM pStage I	OS	2.55 (1.08-6.03)	Univariate
	Chen 2012		OS	2.29 (1.51-3.48)	Univariate
			OS	2.32 (1.51-3.58)	Multivariate
			DFS	2.41 (1.59-3.66)	Univariate
			DFS	2.68 (1.62-3.89)	Multivariate
	Wu 2019		OS	2.97 (1.09-8.12)	Univariate
			DFS	1.87 (0.77-4.51)	Univariate
	Park 2019		DFS	2.43 (1.08-5.50)	Univariate
Breast Cancer	Yue 2016		OS	2.49 (1.15-5.37)	Univariate
			DFS	1.91 (1.01-3.61)	Univariate
Diffuse Large B Cell Lymphoma	Xiao 2008		OS	1.76 (1.3-2.38)	Univariate
		Age (≥50 years)	OS	1.42 (1.02-1.98)	Univariate
		Ann Arbor Stage I-III	OS	1.87 (1.24-2.81)	Univariate
Gastric Cancer	Tan 2018		OS	0.60 (0.40-0.91)	Univariate
		Gender (Male)	OS	0.59 (0.35-0.99)	Univariate
		TNM Stage I-III	OS	0.53 (0.31-0.90)	Univariate
		Lauren_Intestinal	OS	0.50 (0.28-0.90)	Univariate
Colorectal Cancer	Noda 2018	TNM Stage II-III & p53 (-)	DFS	0.28 (0.1-0.76)	Univariate
		TNM Stage II-III & p53 (+)	DFS	1.25 (0.27-5.88)	Univariate
		TNM Stage II-III & p53 (wild-type)	DFS	0.72 (0.41-1.28)	Univariate
		TNM Stage II-III & p53 (mutant)	DFS	1.02 (0.64-1.64)	Univariate
		TNM Stage II-III & p53 (-)	DFS	0.31 (0.11-0.88)	Multivariate
Pancreatic Ductal Adenocarcinoma	Tada 2019		RFS	1.77 (1.06-2.95)	Univariate
Glioma	Gravendeel 2009		OS	1.49 (1.13-1.96)	Univariate
		Gender (Female)	OS	1.99 (1.15-3.44)	Univariate

HR, hazard ratio; OS, overall survival; DFS, disease-free survival.
